# Validation of the Greek Parents’ Evaluation of Aural/Oral Performance of Children (PEACH) Rating Scale v.4 for Greek and Greek–Cypriot Children with Typical Hearing

**DOI:** 10.3390/audiolres15010011

**Published:** 2025-01-24

**Authors:** Paris Binos, Georgios Stavrinos, Loukia Taxitari

**Affiliations:** 1 CIRCLE Laboratory, Department of Rehabilitation Sciences, Cyprus University of Technology, 3036 Limassol, Cyprus; l.taxitari@nup.ac.cy; 2 Department of Education Sciences, European University of Cyprus, 2404 Nicosia, Cyprus; g.stavrinos@external.euc.ac.cy; 3 Department of Psychology, Neapolis University Paphos, 8042 Paphos, Cyprus

**Keywords:** PEACH questionnaire, auditory performance, Greek-speaking children, reliability and validity, developmental trajectories

## Abstract

**Objectives**: This study aimed to adapt and validate the Parents’ Evaluation of Aural/Oral Performance of Children (PEACH) questionnaire for Greek-speaking children aged 1 to 6 years. Key objectives included assessing the PEACH questionnaire’s reliability and validity and determining if children from Greece and Cyprus exhibit similar auditory performance, which would suggest they belong to a comparable population. **Methods**: This cross-sectional study involved 87 children from monolingual Greek-speaking households in Greece (*N* = 38) and Cyprus (*N* = 49), all full-term with typical hearing and no cognitive or language deficits. The children’s ages ranged from 12 to 82 months. The study used an independent samples t-test to compare PEACH Overall Scores between Greek and Greek–Cypriot children. Internal consistency was assessed with Cronbach’s alpha and item-total correlations for each country. Additionally, regression models examined the relationship between PEACH scores and age. **Results**: Greek–Cypriot children had significantly higher PEACH scores (92.09%) than Greek children (86.71%), t(85) = 2.31, *p* = 0.023. The Cronbach’s alpha for the Greek sample was 0.92, indicating a strong internal consistency, while the Greek–Cypriot sample had a lower alpha of 0.79, with item-total correlations ranging from 0.16 to 0.75. Normative curves showed that auditory performance in the Greek sample increased sharply until 40 months, then plateaued until 60 months. In contrast, the Greek–Cypriot sample’s scores rose sharply until 25 months and plateaued by 40 months. **Conclusions**: The Greek-translated PEACH questionnaire demonstrated strong reliability and construct validity for Greek children, consistent with other language adaptations. However, the Greek–Cypriot sample did not achieve similar reliability, and differences in scores suggest potential cultural, linguistic, or environmental factors impacting auditory development. These findings emphasize the importance of regional adaptations in standardized assessments. Further research is recommended to explore factors contributing to these differences for more accurate assessments of Greek-speaking children.

## 1. Introduction

Hearing loss in children is a significant public health concern globally and locally. According to the Centers for Disease Control and Prevention (CDC), the prevalence of hearing loss in newborns in the United States was found to be 1.8 per 1000 screened in their 2020 Annual Early Hearing Detection and Intervention (EHDI) report [1]. Similarly, studies by Stevens et al. have estimated that globally, approximately 1.4% of children between 5 and 14 years old are affected by hearing loss greater than a 35 dB hearing level [2]. In Greece, annual incidences of childhood hearing loss range from 1.5 to 5 cases per 1000 births, translating to roughly 100 children each year based on the country’s birth rate [3]. Cyprus reports a lower prevalence of congenital hearing loss at 1.19 per 1000 births, with the average age at diagnosis being 44 months [4]. In contrast, Greece started its UNHSP in 2009, with a high initial referral rate due to false positives, necessitating retests and further auditory evaluations for failing newborns [5], while cochlear implants are recommended for severe hearing loss around 10 to 12 months of age [6,7]. Objective audiological assessments, however, are limited in describing the effectiveness of hearing aids in children’s daily activities [8]. Many standard questionnaires used in audiological and speech-language assessments are developed primarily in English. When these instruments are adapted for use in different linguistic and cultural contexts, it is crucial to ensure that the translation maintains the validity and reliability of the original version. This process is essential because linguistic nuances and cultural contexts can significantly influence how questions are understood and responded to, potentially affecting the instrument’s ability to measure what it is intended to. An instrument, particularly subjective outcome measures like questionnaires and structured interviews, must accurately measure the specific areas it claims to target. These tools are integral to capturing nuanced data on individual experiences and outcomes, which are often not apparent through objective testing alone. Ensuring the precision and relevance of these subjective measures is crucial for their effective application in diverse clinical and research setting. To ensure that a translated instrument is applicable in diverse cultural and linguistic contexts, validation studies are necessary [9]. For instance, a parental questionnaire designed to assess various listening abilities of children must first be validated in a cohort of parents with typically hearing children [10].

Globally, several questionnaires are extensively utilized in English to assess auditory behavioral performance across varying ages and levels of hearing loss. Notable examples include the Parental questionnaire to evaluate children’s Auditory Behavior in Everyday Life (ABEL) [11], Parent’s Evaluation of Aural/Oral Performance of Children (PEACH) [12,13], Meaningful Auditory Integration Scale (MAIS) [14], Infant–toddler Meaningful Auditory Integration Scale (IT-MAIS) [15], Functional Auditory Performance Indicators (FAPI) [16] and LittlEARS Auditory Questionnaire (LEAQ) [17]. Despite the availability of these tools, in the Greek-speaking world, there are very limited normative data for functional performance measures [18]. The LittlEARS Auditory Questionnaire was initially developed and piloted to assess the auditory behavior of both normal hearing children and hearing-impaired children who receive a cochlear implant or hearing aid before 24 months of age. The PEACH rating scale, on the other hand, is designed for toddlers and preschool children and can be used with older children. Thus, the validation of the PEACH scale in Greek represents a significant contribution by completing a gap in the tools available for evaluating hearing loss and the communicative development of these children.

This study aimed to adopt the PEACH scale into Greek and validate it with typically developing children. It specifically focuses on the validation of PEACH version 4. The PEACH questionnaire, developed by Ching and Hill [13] and later refined by Bagatto and Scollie [19], is a caregiver-reported Patient-Reported Outcome (PRO) tool that assesses hearing performance in daily life. The PEACH questionnaire is available in a Diary and a Rating Scale form; both formats have similar scenarios that concern quiet and noisy real-world environments. Prior research has shown that the normative data obtained from the PEACH Rating Scale [19,20] demonstrate an age-related effect on PEACH scores similar to those derived from the PEACH diary version [13]. It is specifically designed for children aged 3 months to 13 years with sensorineural hearing loss who use hearing aids or cochlear implants. Caregivers report their child’s listening and verbal communication in noise and silence. The original PEACH, as validated by Ching and Hill [13], yielded high reliability with a Cronbach’s alpha score of 0.88 and a meaningful factor structure. The scale has already been adapted into various languages, including Norwegian [10], Swedish [20], Malay [21], Chinese [22], French [23], Portuguese [24], Spanish [25], and Turkish [26], and featured in numerous international studies [27,28].

As Greek is used in two countries, Greece and Cyprus, this study also aimed to validate whether the PEACH scale is suitable for use in both of these Greek-speaking settings. Although the Standard Modern Greek (SMG) is used in Greece, in Cyprus, there is a situation of bidialectalism, where the local dialect, Cypriot Greek, and SMG co-exist. Recent studies show that children in Cyprus might be presenting different developmental trajectories or profiles than children in Greece [29,30,31,32,33,34,35]. This calls for the need to test such tools designed for the evaluation of linguistic, cognitive or communicative development in both countries, as it is unclear whether children being brought up in these countries constitute the same or different population even though they both acquire Greek as their native language.

Internationally recognized auditory inventories and questionnaires include only a few that have been translated and validated into Greek, such as the LEAQ [17]. A validated screening tool is essential for evidence-based practice and is particularly important for the ongoing assessment of Greek and Greek–Cypriot children with hearing loss. The PEACH, however, has not been translated or validated in Greek, either in Greece or Cyprus. Although the adaptation can remain the same, the validation needs to take place in both Greece and Cyprus, as their linguistic environments can be expected to significantly impact the aural/oral performance of children in these regions. Greece, being a largely monolingual society, contrasts sharply with the bidialectal nature of Cyprus, where both Cypriot Greek and Standard Modern Greek are used.

While the original PEACH validation study has included parents of both children with and without hearing loss as old as 19 years old [13], other validation studies of the PEACH in other languages have targeted typical hearing children with ages up to 7 years old [10,19,20,21]. Considering the needs and idiosyncratic characteristics of each country, this study aims to explore the psychometric properties of the PEACH v.4 in Greek and Greek-Cypriot settings, measuring the overall PEACH scores and establishing age norms for children aged 1–6 years using oral communication with typical hearing. We decided to include children only up to 6 years old, as (a) another validated screening questionnaire (the LittleEARS) has already been validated and established in Greek [18], and (b) this demographic represents a critical period for auditory development and intervention. Furthermore, the inclusion of typically hearing children is critical for establishing normative data, which serves as a baseline for evaluating children with hearing impairments. This approach is particularly relevant in clinical and educational contexts, where comparisons to normative benchmarks are essential for assessing auditory performance in children with hearing loss. We are developing a follow-up study that will include also children with hearing loss. 

## 2. Methodology

### 2.1. Participants

The target population was young children between 1 and 6 years of age from Greece and Cyprus with typical hearing as reported by their parents. The final sample adhered to the following inclusion criteria: children have Greek as their mother language; they are being brought up in monolingual home environments, with both parents being Greek or Greek–Cypriot for Greece and Cyprus, respectively; and children were full-term at birth (i.e., born after the 34th week of gestation) and without any known language, cognitive or neural deficits.

Ninety-nine participants from both Greece and Cyprus took part in the study. Seventy-two responses were received via the printed questionnaire, and 27 responses were received via the online questionnaire. Ten cases were excluded from the online questionnaire because they did not meet one or more of the inclusion criteria (i.e., second language at home, child older than 6 years, one parent non-native Greek or non-native Greek–Cypriot). One case in the Greek–Cypriot sample where the parent responded inconsistently was also removed; even though the parent mentioned that the child did not have any hearing loss nor any other disability, in the first screening question (item 1) asking how often the child wears their hearing aid/cochlear implant, they responded “A little”, instead of “Never” as they were instructed to do in case of non-applicability of the item. This case was also an extreme outlier, as the PEACH Overall Score for this case was 2.5% while the mean Overall Score for the country was 90.3%. Moreover, another parent only completed the demographic questions and did not respond to any of the PEACH questionnaires, and these data were also removed from the analysis. All these resulted in a final sample of 87 participants (38 for Greece and 49 for Cyprus). In terms of questionnaire type, there were 71 valid printed questionnaires and 16 valid online questionnaires.

### 2.2. Tools

#### 2.2.1. PEACH Rating Scale

This questionnaire is used to evaluate the effectiveness of a child’s use of hearing in real-world environments. It assesses the child’s ability to engage in conversations, follow instructions, and respond to several types of sounds and voices, both in quiet and noisy environments. Parents are asked to report specific behaviors related to the child’s listening and speaking abilities, such as attention to sounds, clarity of speech, and the ability to recognize sounds. The tool also evaluates the effectiveness of hearing aids or cochlear implants, considering how often and how well these devices are used by the child in daily life.

The PEACH consists of 12 questions rated on a 5-point Likert scale from 0 to 4 (never, rarely, sometimes, often, always) that quantify the child’s performance in different listening situations. Responses are scored to provide a measure of the child’s overall aural/oral performance. Higher scores indicate better performance, while lower scores highlight areas where the child may need additional support or intervention. The questionnaire results in an overall PEACH score and two subscales, a Quiet and a Noise score. Items 3, 5, 7, 9, and 11 are used to form the Quiet subscale, whereas items 4, 6, 8, 10, and 12 are used to form the Noise subscale.

The PEACH Rating Scale was translated into Greek and then back into English using a method known as back translation, which helps maintain the fidelity of the content to the original [19]. Initially, the primary author translated the scale from English to Greek. A bilingual English teacher, who is also fluent in Greek, reviewed this translation for accuracy. Subsequently, a certified speech-language therapist (SLT), who is a native English speaker with proficiency in Greek, translated the text back into English. This individual was briefed about the translation’s purpose but had no prior knowledge of the scale. The first and last authors then reviewed this back-translated version against the original to identify and resolve any discrepancies. The final back-translated text was found to be consistent with the original document. The same questionnaire was produced and used for both Greece and Cyprus. However, the two populations are not necessarily the same regarding language and communication practices. After the translation and the adaptation stage, a preliminary analysis was run to check if the samples from the two countries behaved the same. Greece and Cyprus are different countries, and the latter is characterized by bidialectalism, as described previously. As children are exposed to two different linguistic settings in the two countries, albeit both Greek-speaking, it could be that development differs and they do not belong to the same population. During the preliminary analysis, the parents were told to ask for items/sentences that they did not understand. It was determined that there was no problem it terms of intelligibility, but cultural adaptation steps were not tested.

#### 2.2.2. Demographic Questions

Prior to completing the PEACH Greek questionnaire, parents were also given a demographics questionnaire. These questions assessed several aspects of the child’s development and home family conditions, including the parents’ educational level, the number of children in the family, the birth order of the child in the family, the languages spoken at home, the child’s date of birth, and any hearing, cognitive, language or neural deficits children might have.

### 2.3. Procedure

Data were collected through both printed and online questionnaires. Participants gave written consent, either in writing or through the online survey, by pressing the appropriate button if they agreed to proceed with the study. Before completing the questionnaire, participants were informed about the research’s purpose and the ethical aspects of the study, including anonymity, confidentiality, and the right to withdraw from the survey at any point without penalties. They were also provided with university officers’ contact details in case they had any complaints about the survey. This study was approved by the Cyprus National Bioethics Committee (reference number: ΕΕΒΚ ΕΠ 2024.01.78).

The data collection period lasted approximately twelve weeks. Printed questionnaires were distributed in the Pediatric Clinic of Health Center in Aridea, Pellas in northern Greece, staffed by pediatricians and an ENT specialist, or provided by teachers in two schools in Cyprus. Researchers collected the completed questionnaires and transferred them to electronic form in the laboratory. Printed materials (questionnaires and consent forms) were kept in a locked cabinet with a key that only the researchers had access to. The online questionnaire was created in Google Forms and was distributed in Facebook groups of Greek-speaking mothers, as well as personal acquaintances of the researchers.

### 2.4. Data Analysis

#### 2.4.1. Missing Data

One participating parent did not record a score for item 12 only. As there was only one missing value in the entire dataset, median imputation was used to calculate the score for that missing value.

#### 2.4.2. Reliability Analysis

To assess the internal consistency of the PEACH, we calculated the Cronbach’s alpha, looked at the alpha when item was dropped, and examined the item-total correlation (corrected).

#### 2.4.3. Curve Estimation

Furthermore, to examine the relationship between the PEACH Overall Score and age, we used curve estimation with an inverse regression model.

#### 2.4.4. Construct Validity

Confirmatory Factor Analysis (CFA) was initially conducted to examine the items’ fit to the two predetermined subscales of Quiet and Noise. To assess the model fit of the CFA analyses, we used the Root Mean Square Error of Approximation, the Comparative Fit Index, the Tucker–Lewis Index, and the Standardized Root Mean Square [36]. As these indices indicated a poor model fit, we conducted post-hoc Exploratory Factor Analysis (EFA). The purpose of the EFA was to explore which items grouped together to form distinct factors/subscales independent of the two predetermined subscales. Suitability for allowing us to run an EFA was assessed using the Bartlett’s test of sphericity and the Kaiser–Mayer–Olkin measure. Finally, to determine the number of factors to keep in our EFA, we examined the eigenvalues, scree plots, and parallel analyses results.

#### 2.4.5. Statistical Analysis

The analyses were conducted using R version 4.1.3 via RStudio version 2024.04.1. We first used various R packages to extract the relevant parameters; for Cronbach’s alpha, we used the “psych” package; for the CFA, the “lavaan” and “paran” packages; and for the EFA, the “REdaS” and “nFactors” packages. Finally, we used SPSS version 29 to run curve estimation with inverse regression.

## 3. Results

### 3.1. Preliminary Analysis

During a preliminary analysis, the PEACH Overall Score between the Greek and Greek–Cypriot samples was compared to assess whether the two samples came from the same population. An independent samples t-test revealed that the PEACH Overall Score was significantly higher in the Greek–Cypriot sample (92.09%) compared with the Greek sample (86.71%), t(85) = 2.31, *p* = 0.023. Based on this difference, it was decided that each sample needed to be analyzed separately.

Furthermore, the printed and online versions of the questionnaire were compared to ensure the two versions yielded similar results. Responses from the online versions were only collected from Greek–Cypriot participants, and therefore this analysis only concerned Cyprus. An independent samples t-test showed no significant differences between the two types of questionnaires in the overall PEACH score, t(49) = −0.98, *p* = 0.333. The two versions were therefore collapsed for subsequent analyses in the Greek–Cypriot sample. The reported age of children ranged from 14 to 71 months (median = 44) for the Greek sample and 12 to 82 months (median = 45) for the Greek–Cypriot sample. The ages of the children in the two countries were not significantly different, as shown in an independent samples t-test comparison, t(85) = 0.75, *p* = 0.455. Table 1 presents the descriptive statistics and Cronbach’s alpha for the PEACH Overall Score and each of its two subscales per country.

### 3.2. Consistency and Validity

In the Greek sample, a high internal consistency was found for the PEACH Overall Score and for its Quiet and Noise subscales, with Cronbach’s alpha values ranging from 0.79 to 0.92 (see Table 1). In the Greek–Cypriot sample, a high internal consistency for the Overall Score was found, with a Cronbach’s alpha of 0.79. However, the Quiet and Noise subscales showed a moderate internal consistency, with Cronbach’s alpha values of 0.66 and 0.62, respectively. Table 2 and Table 3 present the results of the item-total correlations and Cronbach’s alpha values when each of the PEACH items was dropped for Greece and Cyprus, respectively. For Greece, all items had moderate to high item-total correlation ranging from 0.53 to 0.79. Similarly, all items showed a very good contribution to the overall reliability of the scale, as none of the items improved the Cronbach’s alpha when deleted.

For the Greek–Cypriot sample, all items except items 3 and 6 had moderate to high item-total correlations ranging from 0.48 to 0.75. Items 3 and 6 had item-total correlations of 0.16 and 0.36, respectively. Similarly, when each of these two items was dropped, the Cronbach’s alpha value was improved, indicating that these items were poorly correlated with the rest of the scale, which in turn reduced the scale’s internal consistency. The rest of the 8 items in the Greek–Cypriot sample did not improve the alpha value when deleted, demonstrating a very good contribution to the overall reliability of the scale.

Table 2 and Table 3 also present the results of the two factor analyses (CFA and EFA) that were conducted for Greece and Cyprus, respectively. In both the Greek sample and the Greek–Cypriot sample, the indices mentioned in Methodology (2.4.4) suggested a poor model fit, and we therefore conducted and interpreted an EFA [37]. Despite the poor CFA model fit for each country sample, the factor loadings are presented in Table 2 (for Greece) and Table 3 (for Cyprus) for reference.

Before running the EFA, we confirmed the suitability of the data for running the analysis using the metrics mentioned in Methodology [38]. We then determined that two factors should be retained in the model, so two factors were retained in the EFA for the Greek sample. An EFA was therefore conducted using promax rotation, as the factors were highly correlated. In Table 2, the factor loadings are presented; as can be seen, they were above the +/−0.32 threshold proposed by Tabachnik and Fidell [39]. We note that items 3, 4, 6, 11, and 12 distinctly loaded onto Factor 2, while items 7, 8, and 9 only loaded onto Factor 1. There were two items (5 and 10) that cross-loaded onto both Factor 1 (0.35 for item 5 and 0.56 for item 10) and Factor 2 (0.42 for item 5 and 0.41 for item 10).

Using the same EFA procedure in the Greek–Cypriot sample as above, the results indicated three factors to be retained in the model. Using the +/−0.32 cutoff as above, items 9, 10, 11, and 12 distinctly loaded onto Factor 1, items 7 and 8 distinctly loaded onto Factor 2, and items 4 and 6 distinctly loaded onto Factor 3. Item 5 cross-loaded onto both Factor 1 and 3, whereas item 3 did not significantly load onto any factor.

### 3.3. Normative Curve

**Figure 1:**
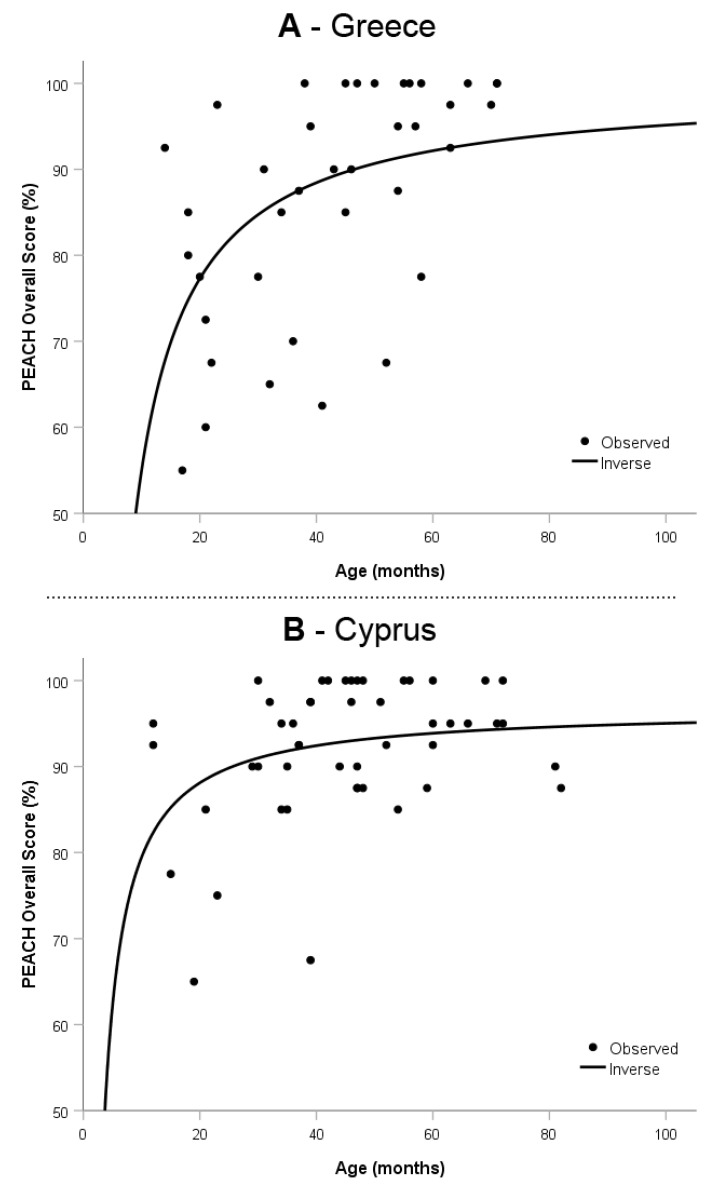
Scatterplot of the PEACH Overall Score (in %) and age (in months) for Greece (**A**) and Cyprus (**B**). PEACH, Parents’ Evaluation of Aural/Oral performance of Children.

A curve estimation with inverted regression model was conducted to assess the relationship between the PEACH Overall Score and the age of the participating children. Figure 1 presents this relationship.

In the Greek sample, the regression line increases rapidly until around 40 months, begins to flatten until about 60 months, and becomes asymptotic beyond 70 months, Beta = −0.41, R^2^_adjusted_ = 0.16, t = −4.15, *p* < 0.001. Therefore, maximum values in the PEACH Overall Score in the Greek sample were recorded between 40 and 70 months. In the Greek–Cypriot sample, the regression line increases sharply until around 25 months, begins to plateau until around 40 months, and becomes asymptotic beyond 70 months, Beta = −0.34, R^2^_adjusted_ = 0.10, t = −2.45, *p* = 0.018. The maximum values in the PEACH Overall Score in the Greek–Cypriot sample were thus observed between 25 and 70 months.

## 4. Discussion

In this study, we adapted the PEACH v.4 questionnaire in Greek and collected data from Greece and Cyprus, marking the first-ever effort to provide this tool for Greek-speaking populations. Data were collected from both countries because of the need to assess its validity and reliability in Greek-speaking populations that exist in different linguistic settings; Greece is a purely monolingual community, while Cyprus is a bidialectal community. In a preliminary analysis, it was observed that the two countries did not exhibit the same behavior, suggesting that children from the two countries come from different populations. This led to the decision to analyze the data separately. This should not come as surprise, as past research has shown differences in how monolingual and bidialectal children develop linguistically and cognitively. Okalidou et al. [40] investigated the phonological development of toddlers in Greece and Cyprus and found that Cypriot children took longer than Greek children to acquire voicing contrasts. Similarly, Antoniou et al. [30] found differences in the executive control abilities between the two populations, with bidialectal and multilingual children showing an advantage compared with their monolingual peers. When evaluating new tools, it is therefore important to ensure that children from the two countries behave similarly before using the data and curves of one population for the other. Using data from Greece for Cyprus could give the wrong impression for children’s development, especially when clinical or educational settings are concerned.

### 4.1. Internal Consistency

Comparing the internal consistency of the Greek PEACH Overall Score in our samples to that of the original PEACH study [13], we note a similarity in the Cronbach’s alpha of our Greek sample to that of the PEACH developers (0.92 in our Greek sample compared to 0.88 in the original Australian study). Nonetheless, the internal consistency in our Greek–Cypriot sample was lower at 0.79. Studies in other languages with children with typical hearing that examined the reliability of the PEACH Overall Score have recorded Cronbach’s alpha values ranging from as low as 0.78 in the (English) Canadian PEACH [20] to as high as 0.98 in the (Mandarin) Chinese PEACH [23]. Our results from both samples fall within this range.

Two items in the Greek–Cypriot sample, items 3 and 6, behaved differently from the rest of the scale, with item 3 being particularly low. This is an unexpected behavior that begs for an explanation if the scale is to be used for children in Cyprus in clinical settings. A more detailed examination of item 3 suggests that the problem may stem from a translation issue. The term “in the absence of noise” (“απουσία θορύβου”) might be challenging to Greek–Cypriots who may not use it as much in their everyday dialect, being a more formal and scholarly expression.

Items 5 and 7 use the same expression, but perhaps after initial exposure to this expression in item 3, they became more accustomed to it. The unexpected behavior of item 6, on the other hand, seems more difficult to explain, especially since items 4 and 8 used the same Greek expression of “in the presence of noise” and did not show inconsistencies in our results. We chose to remove the mention for item 5 here and focused on the comparison with the other items talking about “παρουσία θορύβου” (similar to the earlier comparison for item 3). Both items need to be reviewed more carefully, and more data should be collected for the Greek–Cypriot sample to ensure satisfactory validity and reliability.

### 4.2. Construct Validity

We evaluated the construct validity of the questionnaire by first running a CFA to examine the fit of the items to the two predetermined subscales of Quiet and Noise. However, in neither of our two samples was the fit acceptable, and we thus conducted an EFA to explore the factor structure of the PEACH in each sample. In the Greek sample, the questionnaire was split into two factors: Factor 1 including items 7, 8, and 9 and Factor 2 including items 3, 4, 6, 11, and 12. Items 5 and 10 cross-loaded onto both factors.

Questions in Factor 1 mainly deal with the child’s participation in conversation in both quiet and noise, whereas questions in Factor 2 focus on auditory performance in different situations (including quiet and noisy conditions). There was some overlap between our factor loadings onto each of our two factors and the loadings onto the factors indicated by Ching and Hill in the original PEACH validation study [13]. For instance, items 6, 9, and 10 in both studies distinctly loaded onto Factor 1, while items 3 and 4 distinctly loaded onto Factor 2. Nevertheless, there were also differences between other factor loadings, which may be attributed to the fact that our data were collected using the PEACH rating scale form, whereas in the Australian original validation study, they used the PEACH diary form [13]. In our Greek–Cypriot sample, the EFA indicated three factors to be retained in the model, which contradicted the results of two factors in our Greek sample, as well as other studies that conducted EFAs [10,24]. The validation study of the Spanish PEACH found only one factor when conducting an EFA, which explained 84.6% of the variance [26]. This was the case for their samples of both parents with typical hearing children and parents with hearing-impaired children, but no interpretations of this result were provided. It is possible that the items with a low internal consistency, especially item 3, are causing higher variability in the responses of each subscale, potentially influencing the results obtained in this analysis. Further research addressing the role of these two questions in the Greek–Cypriot sample should be expected to affect and possibly improve construct validity.

### 4.3. Normative Curves

In the Greek sample, the regression line increases sharply until around 40 months and begins to plateau until around 60 months. This finding is not very different from other translated PEACH validation studies [10,21,23]. This suggests a developmental trajectory where initial gains are most significant until around 40 months, followed by a stabilization phase from 60 months onwards. The Greek–Cypriot sample, when compared to our Greek sample, increases sharply at an earlier age (until around 25 months) and flattens out earlier (around 40 months). This pattern indicates that children in the Greek–Cypriot sample reach a developmental plateau sooner than those in the Greek sample, at least as reported by their parents. However, the observed high variability in responses could reflect inherent individual differences in auditory behavior and parental reporting styles. The unequal representation of age groups in the sample could have introduced bias, particularly in the earlier and later age ranges. In addition, the relatively small sample size limits the generalizability of our findings and may have contributed to the observed variability and statistical limitations, which indicate that the regression models only explain a limited proportion of the variance in auditory behavior. We have acknowledged this limitation and included a note that future studies with larger and more diverse samples are needed to improve the explanatory power of the models.

The differences may be also affected by socioeconomic status or linguistic differences between the samples. The different administration methods (printed vs. online) cannot explain the difference between the two samples, as the comparison in our preliminary analysis did not reveal statistical differences between the two administration methods. The investigation of whether individual items differed between the administration methods also showed no significant differences.

### 4.4. Limitations and Further Research

The Greek–Cypriot sample in the current study had consistently lower indices compared with the Greek sample both in terms of reliability and validity in the translated PEACH questionnaire. This may be due to linguistic and cultural differences between Greek and Greek–Cypriot parents. For instance, as pointed out before for item 3, the translation of “quiet” and “noise” in Greek can be loosely translated to “absence of noise” and “presence of noise”, two phrases that may sound more natural for Greek parents compared with Greek–Cypriot parents. Further research is needed using Greek–Cypriot samples before employing the Greek PEACH version in Cyprus. Some modifications in the presentation of PEACH to Greek–Cypriots may be warranted before running new validation studies in Cyprus, namely color coding and emphasizing the distinction between “quiet” and “noise” items.

Due to financial and staff limitations, the questionnaire was not re-administered to assess its test–retest reliability. A very good internal consistency was found, however, in the Greek sample. Further work should administer the Greek-translated PEACH at two time-points to be able to evaluate its test–retest reliability, which may help address some of the issues identified for specific questionnaire items. We aim to run another validation study where test–retest reliability will be included. Other limitations include the lack of formal audiological and health screening, as we relied on parents’ responses regarding the hearing and health status of their children. In addition, only a sample of reportedly typical hearing children was used, which may have influenced the relationship between the PEACH Overall Score and the age presented in our normative curves. This did not allow us to evaluate the discriminatory performance or relevance of the PEACH questionnaire. A future study will be developed where we will expand the scope of our research to include children with hearing impairments, thus assessing the specificity and sensitivity of the PEACH questionnaire as well.

## 5. Conclusions

Overall, the current study demonstrated that the Greek translation of the PEACH questionnaire has a very good reliability (internal consistency), as well as a good construct validity in the Greek sample, comparable to other PEACH validation studies in other languages. Nonetheless, the Greek–Cypriot sample did not exhibit similarly good indices in either the reliability or validity analyses. This may have been due to linguistic differences between the two populations, which beg for a revision of the tool and collection of more data. While the Greek version of PEACH can be used in Greece to identify children at risk of hearing ability, in Cyprus, further validation studies are required. For both countries, further research is required using samples of parents with hearing-impaired children.

## Figures and Tables

**Table 1:** Descriptive statistics and Cronbach’s alpha for PEACH scores and age by country.

Greece (*N* = 38)				
	Overall	Quiet	Noise	Age
Mean	86.71	88.29	85.13	42.53
SD	13.48	12.80	14.77	17.03
Cronbach’s alpha	0.92	0.79	0.87	-
Cyprus (*N* = 49)				
	Overall	Quiet	Noise	Age
Mean	92.09	93.37	90.82	45.29
SD	8.09	8.68	8.92	17.02
Cronbach’s alpha	0.79	0.66	0.62	-

PEACH, Parents’ Evaluation of Aural/Oral performance of Children; SD, Standard Deviation.

**Table 2:** Results of reliability analysis, CFA, and EFA for Greece.

Greece						
Item	ITC Corrected	*α* If Item Deleted	CFA		EFA	
			Quiet	Noise	Factor 1	Factor 2
3	0.53	0.92	0.53			0.82
4	0.74	0.91		0.72		0.51
5	0.72	0.91	0.70		0.35	0.42
6	0.79	0.90		0.77		0.56
7	0.76	0.91	0.74		0.86	
8	0.72	0.91		0.73	1.12	
9	0.77	0.91	0.74		0.65	
10	0.89	0.90		0.88	0.56	0.41
11	0.72	0.91	0.66			0.75
12	0.74	0.91		0.71		0.79
Variance explained			46.5%	58.6%	32.3%	30.7%

CFA, Confirmatory Factor Analysis; EFA, Exploratory Factor Analysis; ITC, Item-Total Correlation.

**Table 3:** Results of reliability analysis, CFA, and EFA for Cyprus.

Cyprus							
Item	ITC Corrected	α If Item Deleted	CFA		EFA		
			Quiet	Noise	Factor 1	Factor 2	Factor 3
3	0.16	0.82	0.12				
4	0.48	0.78		0.35			0.53
5	0.61	0.78	0.52		0.42		0.43
6	0.36	0.80		0.20			0.92
7	0.75	0.75	0.76			0.76	
8	0.62	0.77		0.62		0.94	
9	0.63	0.76	0.66		0.66		
10	0.63	0.76		0.68	0.63		
11	0.73	0.75	0.78		0.94		
12	0.56	0.77		0.55	0.61		
Variance explained			38.3%	26.3%	23.8%	16.9%	13.0%

CFA, Confirmatory Factor Analysis; EFA, Exploratory Factor Analysis; ITC, Item-Total Correlation.

## Data Availability

Data are unavailable due to ethical restrictions.
